# 13 Statewide Prehospital Routing of Burn Injuries Reduces Patient Length of Stay

**DOI:** 10.1093/jbcr/irac012.017

**Published:** 2022-03-23

**Authors:** Randy D Kearns, Tracee Short, Joseph Barrios, Kevin Sittig, Paige B Hargrove, Chris W Hector, Jeffrey E Carter

**Affiliations:** University of New Orleans, New Orleans, Louisiana; Baton Rouge General, Baton Rouge, Louisiana; Our Lady of Lourdes, Lafayette, Louisiana; LSU Shreveport, Shreveport, Louisiana; Louisiana Emergency Response Network, Baton Rouge, Louisiana; Louisiana Emergency Response Network, Baton Rouge, Louisiana; University Medical Center- New Orleans, New Orleans, Louisiana

## Abstract

**Introduction:**

In the U.S., traumatic injuries are the leading cause of death before age 45 and have a significantly lower mortality if routed to a verified trauma center. Burn injuries are included in trauma statistics and represent 1.1 million injured people annually seeking medical assistance. Routing of burn injuries to ABA-recognized burn centers has yet to be assessed as it has in trauma injury. Our goal was to examine the impact of prehospital routing of burn injuries on hospital length of stay, mortality, and potential costs of care through a statewide care coordination center, the Louisiana Emergency Response Network.

**Methods:**

Our study is a retrospective statewide analysis of burn injuries from 01/01/2017 thru 12/31/2019 using the Louisiana Hospital Inpatient Discharge Database. Routing of burn patients was implemented in 2018 using the ABA burn referral criteria. Data included: total admissions with primary burn diagnosis, region, discharge status, length of stay, and raw mortality by region and state. Descriptive and comparative statistics were performed to assess the impact of routing of burn injured patients. Cost analysis was performed using Louisiana Medicaid per diem rates from 2021 at $1,907.92/day.

**Results:**

1,288 patients were treated in Louisiana during the study period with 855 post-routing and 433 pre-routing. The mean length of stay was reduced from 11.84 days in 2017 to 8.82 days in 2018 (*p* value=0.0988) with a potential savings of 761 inpatient care days or $2.17 million. Overall mortality across the state was unchanged except in the highest volume region where it dropped from 7.9% in 2017 to 3.6% in 2019 (54%).

**Conclusions:**

Burn injuries meeting ABA referral criteria are a form of time-sensitive trauma. This study marks the first analysis pre and post implementation of routing for burn injuries by a statewide care coordination center. Our study demonstrates improvement in length of stay and mortality but a continued need to examine other contributing factors such as severity of injury and concomitant trauma.

